# ACSL4 promotes hepatocellular carcinoma progression via c-Myc stability mediated by ERK/FBW7/c-Myc axis

**DOI:** 10.1038/s41389-020-0226-z

**Published:** 2020-04-29

**Authors:** Junru Chen, Chaofeng Ding, Yunhao Chen, Wendi Hu, Yuejie Lu, Wenxuan Wu, Yanpeng Zhang, Beng Yang, Hao Wu, Chuanhui Peng, Haiyang Xie, Lin Zhou, Jian Wu, Shusen Zheng

**Affiliations:** 1 Division of Hepatobiliary and Pancreatic Surgery, Department of Surgery, First Affiliated Hospital, School of Medicine, Zhejiang University, 310003 Hangzhou, China; 2 NHC Key Laboratory of Combined Multi-organ Transplantation, 310003 Hangzhou, China; 3 Key Laboratory of the Diagnosis and Treatment of Organ Transplantation, CAMS, 310003 Hangzhou, China; 4 Key Laboratory of Organ Transplantation, 310003 Hangzhou, Zhejiang Province China; 5 Zhejiang Provincial Research Center for Diagnosis and Treatment of Hepatobiliary Diseases, Hangzhou, 310003 China

**Keywords:** Liver cancer, Oncogenes

## Abstract

Hepatocellular carcinoma (HCC) is a highly heterogeneous, multigene-driven malignant tumor. Long chain acyl-CoA synthetase 4 (ACSL4), an enzyme has pivotal roles in arachidonic acid (AA) metabolism. However, its function and the underlying molecular mechanisms in HCC are still not fully elucidated. Here, we identified ACSL4 as a novel marker for AFP high subtype HCC through transcriptome profiling. ACSL4 was frequently upregulated in HCC samples and associated with poor prognosis. Functionally, ACSL4 knockdown resulted in decreased cell growth, whereas ectopic ACSL4 expression facilitated tumor formation in vitro and in vivo. Mechanistically, ACSL4 stabilized the oncoprotein c-Myc through ubiquitin–proteasome system in an ERK/FBW7-dependent manner. Cell growth ability mediated by ACSL4 elevation was partly attenuated by c-Myc depletion using siRNA or its inhibitor 10058-F4. In contrast, the effects of ACSL4 silencing were partially reversed by c-Myc overexpression via FBW7 knockdown. Clinically, ACSL4 expression was positively correlated with c-Myc in HCC. In conclusion, ACSL4 is a novel marker for AFP high subtype HCC. Our data uncovered a new mechanism by which ACSL4 promotes HCC progression via c-Myc stability mediated by ERK/FBW7/c-Myc axis and could be a valuable prognostic biomarker and a potential therapeutic target in HCC.

## Introduction

Hepatocellular carcinoma (HCC) accounts for 75–85% of primary liver cancer^1^, and ranks the fourth leading cause of cancer-related death worldwide due to lack of effective diagnostic markers for early detection and the high recurrence rate^2^. Currently, surgical resection or liver transplantation are the most effective treatment for HCC, but most patients with HCC are diagnosed at advanced stage, which are not suitable for surgery^3^. Until now, sorafenib and lenvatinab are FDA approved first-line systemic treatment for advanced HCC, with limited efficacy in individuals^4,5^. However, the molecular mechanisms underlying HCC progression are still not fully understood. Thus, identification of novel causative genes and molecular pathways underlying HCC carcinogenesis may provide important clues for HCC treatment.

Uncontrolled proliferation is a hallmark of cancer. In HCC, one of the major types of molecular classification is a proliferation class^6^. The proliferation class of HCC was associated with HBV infection, high alpha-fetoprotein (AFP) levels, poor differentiation and worse outcomes. AFP as a critical serum biomarker of HCC, represents a clinical aggressive phenotype. A recent randomized control trial confirmed the survival benefit of ramucirumab on a subtype of HCC patients whose AFP concentrations were more than 400 ng/ml^7^. Moreover, the proteomics of early-stage HCC revealed the patients with high AFP concentrations displayed additional upregulation in immunity-related pathways and cell adhesion molecules^8^. These studies provide evidence that overexpression of AFP has been associated with a distinct phenotype of HCC, as it has a distinct biology, and requiring distinct treatment strategy. Therefore, uncovering the molecular features of AFP high subtype of HCC is urgently needed for personalized cancer therapy.

Long chain fatty acyl-CoA ligase 4 (ACSL4) is one of Acyl-CoA synthetases (ACS) family of members that convert fatty acid to fatty acyl-CoA esters, and has a substrate preference for arachidonic acid (AA)^9^. Under normal conditions, ACSL4 is abundant in steroidogenic tissues, whereas it is poorly expressed in the gastrointestinal system, including liver^10^. So far, it has been reported that the ACSL4 dysregulation is related to a great number of malignant tumors such as, breast cancer, colon adenocarcinoma and gastric cancer^11–13^. ACSL4 exhibits tumor-suppressing activity in gastric cancer^13^, while it has important oncogenic roles in contributing to breast and prostate cancer initialization and metastasis^11,14^, suggesting the relationship between ACSL4 and cancer development is complex. Nevertheless, the oncogenic functions of ACSL4 and its underlying molecular mechanisms in HCC remain unclear to date.

In the present study, we identified ACSL4 as a novel marker for AFP high subtype HCC through transcriptome profiling, and uncovered a crucial role of ACSL4 in mediating c-Myc stability in HCC. Upregulation of ACSL4 correlates with unfavorable prognosis in HCC individuals. Mechanistically, ACSL4 can stabilize c-Myc expression at a post-transcription level in an ERK/FBW7-dependent manner, resulting in elevated tumor cell proliferation and enhanced tumor progression. Our findings highlight the potential for therapeutics aimed at inhibiting this ACSL4/c-Myc cascade pathway.

## Materials and methods

### HTA 2.0 transcriptome microarray assay

Total RNA was isolated with Trizol from randomly group selected HCC and adjacent non-tumor tissues with indicated AFP values. Biotinylated cDNAs were prepared according to the standard Affymetrix protocol from 250 ng total RNA by using Ambion® WT PLUS Reagent Kit. Following labeling, 5.5 μg of cDNA were hybridized on GeneChip Human Transcriptome Array 2.0 in Hybridization Oven 645. GeneChips were washed and stained in the Affymetrix Fluidics Station 450. GeneChips were scanned by using Affymetrix® GeneChip Command Console (AGCC) software which installed in GeneChip® Scanner 3000. The data were analyzed with Robust Multichip Analysis (RMA) algorithm using Affymetrix default analysis settings and global scaling as normalization method. Values presented are log_2_ RMA signal intensity.

### Patients and HCC tissue samples

Tumor tissues and matched normal tissues were collected from HCC patients who received curative surgery. Two independent cohorts of HCC patients were enrolled in this study, total 30 pairs of primary HCC tumor tissues and the matched normal tissues, and 87 HCC tumor samples were collected from the First Affiliated Hospital, Zhejiang University School of Medicine, Zhejiang, China. For the first cohort (cohort 1), matched fresh specimens of HCC tissues and adjacent non-tumorous liver tissue were collected to test the expression of ACSL4 by qRT-PCR and immunohistochemical staining. In addition, 10 paired HCC samples in cohort 1 were randomly selected for western blotting analysis. For the second cohort (cohort 2), 87 HCC tumor samples with clinicopathological and follow-up data were used for tissue microarray. This research was approved by the Ethical Review Committee of this hospital. Written informed consent was received according to the guidelines of the Declaration of Helsinki.

### Immunohistochemistry (IHC)

The immunohistochemistry analysis was performed as we previously reported^15^. The primary antibodies against ACSL4 (ab155282) (Abcam) and c-Myc (10828-1-AP) (Proteintech) were used at concentrations of 1:1000 and 1:200, respectively. IHC intensity scores were defined as follows: 1 (weak), 2 (medium), 3 (strong) and 4 (very strong). Percentage of positive cells was scored as: 1 (0–25%), 2 (26–50%), 3 (51–75%) and 4 (>75%). Overall score was determined by multiplying the intensity score by positive cell percentage score. Overall score more than 8 (≥8) was defined as high expression, whereas the others represented low expression. All stained slides were reviewed independently by two pathologists who were unaware of the patient outcomes.

### Cell culture and reagents

All cell lines were obtained from the Cell Bank of the Shanghai Institutes of Biological Sciences, Chinese Academy of Sciences. Cells were maintained in Minimum Essential Media (MEM, Gibco, USA) supplemented with 10% fetal bovine serum (Biological Industries, Israel). Cells were cultured in a humidified atmosphere with 5% CO_2_ at 37 °C. Exponentially growing cells were used for subsequent experiments.

MG132, cycloheximide (CHX) and 10058-F4 were purchased from MCE; Triacsin C was from Bio-Techne; Z-VAD-FMK was from APE×BIO, and SCH772984 was from Selleck Chemicals.

### RNA extraction and quantitative real-time PCR (qRT-PCR)

Total RNA was extracted from cell lines and fresh tumor tissues using TRIzol Reagent (Thermo Scientific, USA) according to the manufacturer’s protocol. For the mRNA analysis, the TaKaRa PrimeScript RT Reagent Kit (TaKaRa, Japan) and the Bio-Rad QX100 Droplet Digital PCR system were used to conduct real-time quantitative PCR analysis. GAPDH was used as internal control. Relative expression levels of each gene were calculated according to the 2^−△△Ct^ method. All premiers were obtained from Tsingke Biological Technology (Beijing, China). Specific Primer sequences were listed as follows:

ACSL4, 5′-CATCCCTGGAGCAGATACTCT-3′ (forward) and

5′- TCACTTAGGATTTCCCTGGTCC-3′ (reverse);

c-Myc, 5′- GGCTCCTGGCAAAAGGTCA-3′ (forward) and

5′- CTGCGTAGTTGTGCTGATGT-3′ (reverse);

GAPDH, 5′- GGAGCGAGATCCCTCCAAAAT-3′ (forward) and

5′- GGCTGTTGTCATACTTCTCATGG-3′ (reverse).

### Western blotting (WB)

Total protein was extracted from tissues or cells using ice-cold radioimmunoprecipitation assay (RIPA) buffer containing protease and phosphatase inhibitors (Cell Signaling Technology, USA). Protein was quantified using the BCA Protein Assay Kit (Thermo Scientific, USA) and boiled for 10 min at 99 °C. Protein samples were separated using sodium dodecyl sulfate-polyacrylamide gel electrophoresis (SDS-PAGE), transferred to polyvinylidene fluoride (PVDF) membrane (Merck Millipore, Burlington, MA, USA), and blocked with 5% skim milk in Tris-buffered saline containing Tween-20 (TBST) at room temperature for 1 h. Membranes were probed with primary antibodies at 4 °C overnight with gentle rocking, followed by incubation with horseradish peroxidase (HRP)-conjugated secondary antibodies for 1 h at room temperature before visualization by ECL Kits (Thermo Scientific, USA). The integrated density of the band was quantified using Image Lab software (Bio-Rad, Hercules, CA, USA). In total, primary antibodies included those for ACSL4 (ab155282), c-Myc (ab32072), c-Myc (phospho-T58, ab185655), c-Myc (phospho-S62, ab185656), FBW7 (ab109617, ab171961), Lamin B1 (ab229025), caspase-3 (ab184787) (Abcam, UK), Flag (A00187) (Genscript, China), SKP2 (#2652), Bax (#5023), Bcl-2 (#15071), PARP (#9532), CDK2 (#2546), CDK4 (#12790), CDK6 (#13331), cyclinD1 (#2922), ERK (#4695), p-ERK (#9101) (Cell Signaling Technology, USA) and GAPDH (10494-1-AP) (Proteintech, China). GAPDH was used as a loading control.

### SiRNA and plasmids

The siRNA target sequences used in this study are as follows: ACSL4 siRNA-1, 5′- GGGAGUGAUGAUGCAUCAUAGCAAU-3′; ACSL4 siRNA-3, 5′- GGCUUCCUAUCUGAUUACCAGUGUU-3′; c-Myc siRNA, 5′-GGAAACGACGAGAACAGUU-3′; FBW7 siRNA, 5′-GGGACATACAGGTGGAGTA-3′; SKP2 siRNA, 5′-GCCTAAGCTAAATCGAGAGAA-3′. The siRNAs for ACSL4 were purchased from Invitrogen (Thermo Scientific, USA), others were all synthesized by GenePharma (Shanghai, China). The transfection concentration was 10 nM. Empty vector, pcDNA3.1-ACSL4 were ordered from GeneCopoeia for overexpression assays. The cells were transiently transfected with jetPRIME® (Polyplus-transfection SA, France) following the manufacturer’s instructions. Transfection efficiency was determined by qRT-PCR and immunoblot analysis.

### Lentivirus transfection and stable cell clone establishment

Lentiviral-based small hairpin RNA (shRNA) targeting ACSL4 (target sequence: AUUGCUAUGAUGCAUCAUCACUCCC) were purchased from GeneChem (Shanghai, China). Lentiviruses were infected into HCC cells with an MOI 10 plus 5 mg/ml polybrene (Genechem). After infection for 48 h, cells were selected for 2 weeks using puromycin (2 μg/ml, Selleck Chemicals) to produce a stable cell line for subsequent assays.

### Cell counting kit-8 (CCK-8) assay

Cell proliferation viability was assessed by CCK-8 assay (Dojindo Laboratories, Japan). Cells were seeded into 96-well plates at a density of 3 × 10^3^ cells per well with 200 μl of culture medium. With supernatant removed, a total of 10 μl of CCK-8 reagent in 100 μl medium was added to each well at the indicated time points (6, 24, 48, 72, 96 h). The plates were incubated in dark at 37 °C for 2 h, and the absorbance at 450 nm was detected with a microplate reader (BioTek, USA). The experiments were performed in triplicate.

### Colony-formation assay

Cells (1 × 10^3^ cells per well) were plated in each well of a 6-well cell culture plate. After cultured for 2 weeks, the colonies on the plates were fixed in 4% paraformaldehyde, stained with 1% crystal violet, and the numbers of colonies were then counted to evaluate cell proliferation. The experiments were conducted in triplicate.

### 5-ethynyl-2′-deoxyuridine (EdU) incorporation assay

EdU incorporation assay was performed using Cell-Light™ EdU Apollo488 In Vitro Imaging Kit (RiboBio) according to the manufacturer’s instructions. Briefly, cells were seeded into at the density of 1 × 10^5^ cells per well and incubated overnight. Then the cells were incubated with EdU for 2 h at 37 °C and fixed in 4% paraformaldehyde. After permeabilization with 0.5% Triton X-100 in phosphate-buffer saline (PBS), the cells were reacted with 1× Apollo reaction cocktail for 30 min. Subsequently, the nuclei were labeled with Hoechst 33342 for 30 min and imaged on a fluorescence microscope. All studies were conducted in triplicates.

### Cell cycle and apoptosis analysis by flow cytometry

For cell-cycle analysis, cells were trypsinized and fixed with 75% ethanol overnight at −20 °C for permeabilization. After being washed with phosphate buffered saline (PBS) and resuspended with 300 μl DNA staining solution (Multiscience, China) at room temperature for 30 min. The cell-cycle analysis was detected via a flow cytometry (FACS LSRII, BD Bioscience, USA). At least 10,000 events were recorded for each sample. The cell-cycle data were analyzed by Mod-Fit LT 5.0 software (Verity Software House, USA).

Cell apoptosis was detected by using the Annexin V-FITC/ Propidium Iodide (PI) Apoptosis Detection Kit (BD Biosciences) according to the manufacturer’s instruction. In brief, cells (1 × 10^6^) were trypsinized and centrifuged, then suspended with 400 μl of binding buffer and incubated with 5 μl Annexin V-FITC and PI in the dark condition for 30 min before being analyzed by flow cytometry (BD Biosciences). At least 10,000 events were recorded for each sample. The cell apoptosis data were analyzed by Flowjo V10 software (Tree Star, San Francisco, CA, USA).

### Cellular fractionation assay

The HCC cells were fractionated by Nuclear and Cytoplasmic Protein Extraction Kit (Beyotime) to isolate the nuclear and cytoplasmic proteins according to the manufacturer’s instructions. These fractions were prepared and subjected to western blot assay.

### Ubiquitination assays

Cells were incubated with 15 μM MG132 for 6–8 h. Total proteins were extracted using RIPA and PMSF. IP assay was then conducted using antibody against c-Myc (Proteintech, China). Western blotting was performed to assess the ubiquitination level of the immunoprecipitated proteins using ubiquitin antibody (# 3933) (Cell Signaling Technology, USA). The protein levels of c-Myc in input and ACSL4, c-Myc, p-c-Myc (S62), FBW7 and SKP2 in whole-cell lysates (WCL) were examined using western blotting assays.

### Immunofluorescence (IF)

Indicated cells were seeded on the slides at the density of 1 × 10^5^ cells per well and incubated overnight. After the incubation, the cells were washed with PBS, fixed with 4% paraformaldehyde solution, permeabilized with 0.1% Triton X, and blocked with bovine serum albumin (BSA) for 1 h. Then cells were incubated with c-Myc (GTX103436) (GeneTex, 1:100) primary antibody at 4 °C overnight. Subsequently, the slides were washed with PBS and treated with secondary antibodies, then counterstained with DAPI, and visualized with a confocal microscopy.

### Xenograft studies in nude mice

Four-week-old male BALB/c nude mice were purchased from Shanghai Experimental Animal Center of Chinese Academic of Sciences (Shanghai, China). A total of 4 × 10^6^ Huh7 cells or 5 × 10^6^ SMMC-7721 cells suspended in 100 μl PBS were subcutaneously injected into the flanks of nude mice (randomly selected, six mice per group). The subcutaneous tumor size was calculated and recorded every four days using the following equation: tumor volume = (length × width^2^)/2. Four weeks after injection, mice were killed and the tumors were surgically dissected, collected and embedded in paraffin, followed by H&E (hematoxylin and eosin) and IHC staining. The harvested tumors were also used for western blot with antibodies against ACSL4 and c-Myc. All animal experiments were approved by the Ethics Committee for Laboratory Animals of the First Affiliated Hospital, Zhejiang University School of Medicine, Zhejiang, China.

### Statistical analysis

Statistical analyses were performed using SPSS 22.0 (SPSS, Chicago, IL, USA) and Prism 7.0 (GraphPad Software, La Jolla, CA, USA) software. Data are presented as the mean ± SD of at least three independent experiments. Quantitative data were compared using two-sided Student’s *t*-test or Wilcoxon matched-pairs test. Categorical data were analyzed by the Fisher exact test. Overall survival (OS) or recurrence-free survival (RFS) in relation to ACSL4 expression was evaluated by the Kaplan–Meier survival curve and the log-rank test. Relative risks (RR) of prognostic factors associated with ACSL4 expression and other predictor variables were estimated by using a univariate Cox proportional hazards regression model and multivariate Cox model. A two-tailed *P* < 0.05 was considered statistically significant.

## Results

### ACSL4 is identified as a novel marker for HCC with high AFP

To identify driver oncogenes associated with high AFP concentration in HCC, we performed transcriptomic microarray analysis in paired cancer samples. We selected surgically resected primary HCC tissues and paired non-tumor liver tissues from six cases of patients who had not experienced prior chemotherapy or radiotherapy. Clinical features were listed in Supplementary Table 1.

**Fig. 1:**
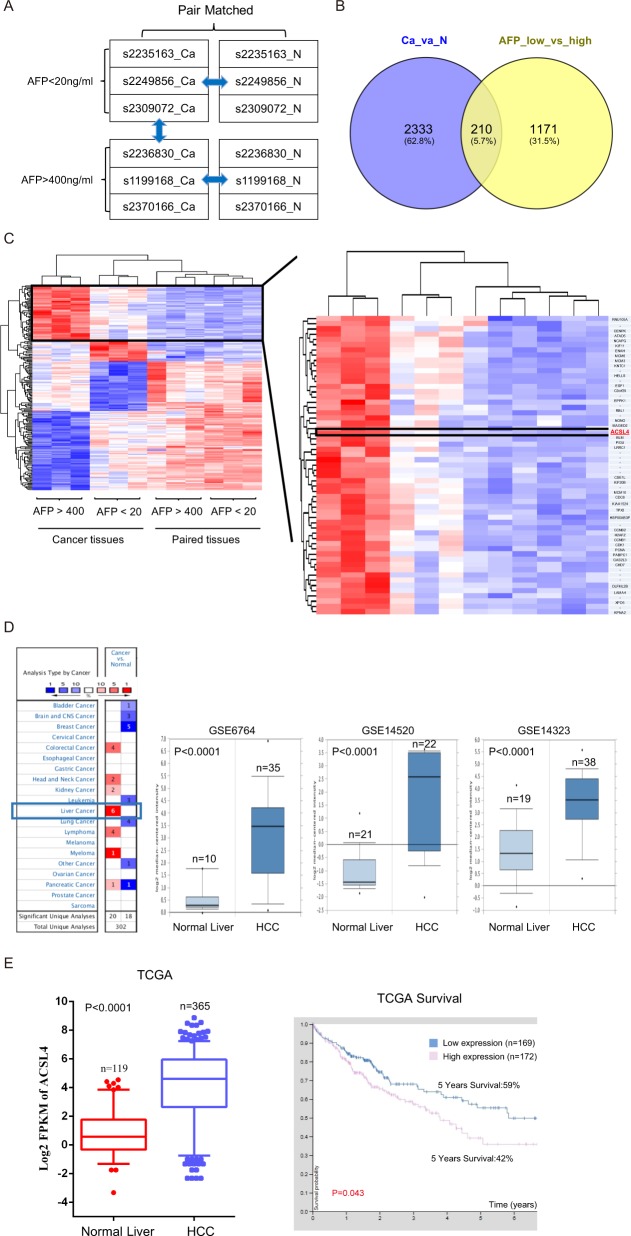
ACSL4 is identified as a novel marker for HCC with high AFP. **a** Schematic diagram of transcriptome array design. **b** Commonly involved transcripts in HCC- and AFP-related genes. **c** Heatmap of 12 samples included in this study. **d**, **e** Box plots indicated ACSL4 mRNA expression in HCC from three gene expression datasets (GSE6764, GSE14520, GSE14323) and TCGA database, respectively. Comparisons were analyzed by Wilcoxon matched-pairs test, and Kaplan–Meier survival.

Two groups were divided according to preoperative AFP levels: AFP^low^ group (AFP < 20 ng/ml, *n* = 3) and AFP^high^ group (AFP > 400 ng/ml, *n* = 3) (Fig. 1a). Transcriptomic analysis was performed and identified 2543 transcripts from six paired tissues, as well as 1381 transcripts from AFP low versus high patients. Interestingly, 210 transcripts were commonly involved in HCC related genes and AFP-related genes (Fig. 1b). ACSL4 is one of the top outlier genes, which exhibits the high expression levels both in AFP^high^ group and HCC tissues (Fig. 1c and Supplementary Table 2), suggesting a novel marker for AFP^high^ patients. Targeting this transcript may improve beneficial in clinical settings.

To investigate the expression pattern of ACSL4 in HCC, we first analyzed multiple publicly available gene expression datasets (GSE6764, GSE14520, GSE14323 and The Cancer Genome Atlas [TCGA]). We noticed that ACSL4 mRNA expression was remarkably upregulated in HCC (Fig. 1d, e). Furthermore, high ACSL4 mRNA level was significantly associated with poor prognosis by analyzing TCGA dataset that contains 341 HCC patients (Fig. 1e).

### ACSL4 expression is upregulated in HCC and associated with poor outcomes

**Fig. 2:**
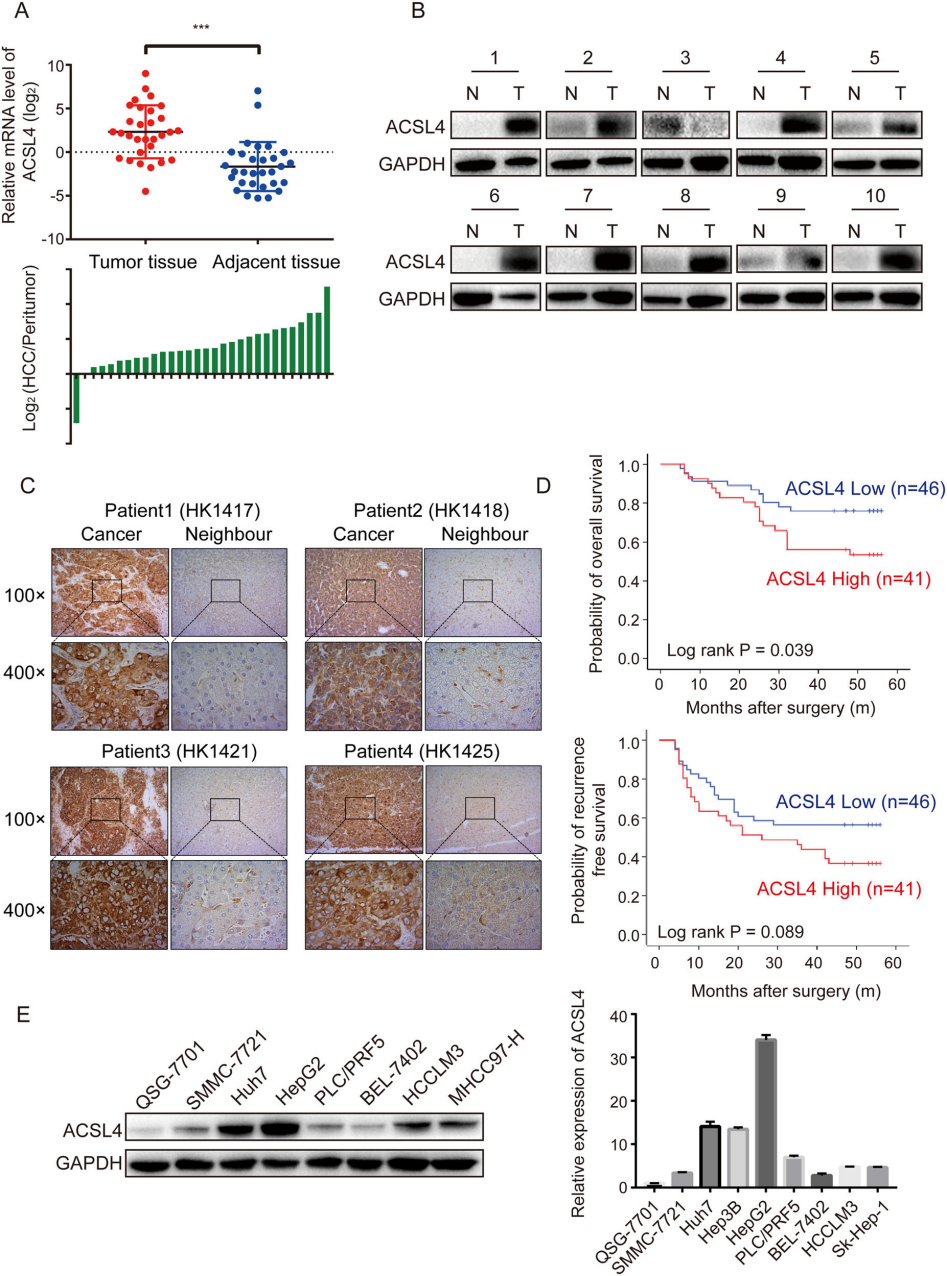
ACSL4 expression is upregulated in HCC and associated with poor outcomes. **a** Expression levels of ACSL4 mRNA in 30 paired HCC specimens were investigated by qRT-PCR (upper). Wilcoxon matched-pairs test was performed. Comparison of ACSL4 mRNA expression between HCC and corresponding peritumor tissues is shown as a log_2_-fold change (lower). ***p < 0.001. **b** The protein expression of ACSL4 was examined in 10 pairs of HCC tumor tissues (T) and corresponding neighbor tissues (N). **c** The expression of ACSL4 in paired HCC samples from cohort 1 was confirmed by immunohistochemical staining. Representative examples of ACSL4 staining are shown. **d** Kaplan–Meier survival analyses were conducted to evaluate the influence of ACSL4 on overall survival and recurrence-free survival. **e** The expression levels of ACSL4 protein and mRNA in HCC cell lines and normal liver cell line QSG-7701 were investigated using western blotting and qRT-PCR.

To determine the role of ACSL4 in HCC, we examined the mRNA expression level of ACSL4 in 30 human HCC tissues and their adjacent peritumor specimens (cohort 1) by qRT-PCR. In accordance with GEO and TCGA databases, ACSL4 mRNA level was markedly upregulated in HCC tissues (Fig. 2a). Moreover, increased protein level of ACSL4 was verified using western blotting in ten paired HCC tissues randomly obtained from cohort 1 (Fig. 2b). Immunohistochemistry also confirmed the upregulated protein level of ACSL4 in HCC tissues in cohort 1 (Fig. 2c). Therefore, ACSL4 can serve as a biomarker overexpressed in human HCC.

To explore whether ACSL4 expression was associated with the clinicopathological significance or patient survival, we determined ACSL4 expression in 87 pairs of HCC tissues (cohort 2) by IHC followed with independent visual scoring of two pathologists. Baseline of Clinicopathological features were listed in Supplementary Table 3. Patients from cohort 2 were then divided into a high expression group (*n* = 46) and a low expression (*n* = 41) group according to the IHC scores of ACSL4. Significant correlations between ACSL4 expression and serum AFP level were observed (*p* = 0.004, Table 1), Kaplan–Meier analysis revealed that patients with high ACSL4 expression had poor overall survival and recurrence-free survival (Fig. 2d). Furthermore, multivariate Cox regression analysis revealed that ACSL4 expression was an independent prognostic factor for poor overall survival in patients with HCC (HR: 2.434, 95% CI: 1.149–5.153, *p* = 0.020, Table 2). Although no statistical significance of ACSL4 expression for recurrence-free survival (HR: 1.663, 95% CI: 0.921–3.004, *p* = 0.092, Table 3) was indicated, patients with high expression levels of ACSL4 were demonstrated a trend for HCC recurrence.

**Table 1:** Clinicopathological correlation of ACSL4 expression in human HCC.

Variables	Tumor ACSL4 expression		
	Low	High	*p*-value^a^
Age			
≤50 years	19 (47.5%)	21 (52.5%)	0.354
>50 years	27 (57.4%)	20 (42.6%)	
Gender			
Female	5 (62.5%)	3 (37.5%)	0.567
Male	41 (51.9%)	38 (48.1%)	
HBsAg status			
Negative	9 (52.9%)	8 (47.1%)	0.995
Positive	37 (52.9%)	33 (47.1%)	
Cirrhosis			
Absent	5 (55.6%)	4 (44.4%)	0.865
Present	41 (52.6%)	37 (47.4%)	
Tumor encapsulation			
Complete	20 (50%)	20 (50%)	0.620
No complete	26 (55.3%)	21 (44.7%)	
Tumor size			
≤5 cm	29 (47.5%)	32 (52.5%)	0.127
>5 cm	17 (65.4%)	9 (34.6%)	
Tumor number			
Single	43 (55.8%)	34 (44.2%)	0.123
Multiple	3 (30%)	7 (70%)	
Edmondson–Steiner grade			
I–II	35 (54.7%)	26 (45.3%)	0.572
III–IV	11 (47.8%)	12 (52.2%)	
PVTT			
Absent	32 (50.8%)	31 (49.2%)	0.529
Present	14 (58.3%)	10 (41.7%)	
Alpha-fetoprotein level			
≤20 ng/ml	25 (71.4%)	10 (28.6%)	0.030
>400 ng/ml	14 (45.2%)	17 (54.8%)	

^a^*p*-values were derived with a two-tailed Pearson chi-square test.

**Table 2:** Cox univariate and multivariate analysis of predictors of overall survival following hepatectomy.

Variables for overall survival	Univariate analysis	*p*-value	Multivariate analysis	*p*-value
	HR (95% CI)		HR (95%CI)	
Age, year (>50 versus ≤50)	1.330 (0.640–2.762)	0.444		
Gender (male versus female)	3.157 (0.430–23.193)	0.258		
HBsAg (positive versus negative)	0.918 (0.375–2.247)	0.852		
Cirrhosis (present versus absent)	1.754 (0.418–7.365)	0.443		
Tumor encapsulation (complete/no)	2.041 (0.955–4.364)	0.066	2.195 (1.024–4.707)	0.043
Tumor size, cm (>5 versus ≤5)	1.985 (0.963–4.089)	0.063	2.217 (1.070–4.592)	0.032
Tumor number (multiple versus single)	1.659 (0.634–4.342)	0.302		
Edmondson–Steiner grade (I–II / III–IV)	2.073 (0.998–4.306)	0.051	NA	NA
Vascular invasion (yes versus no)	2.167 (1.051–4.467)	0.036	NA	NA
Preoperative AFP level, ng/ml (>400 versus ≤400)	1.120 (0.533–2.354)	0.765		
TNM stage (I/II–III)	1.707 (0.832–3.500)	0.144		
ACSL4 expression (high versus low)	2.136 (1.016–4.492)	0.045	2.434 (1.149–5.153)	0.020

*AFP* alpha-fetoprotein, *HR* hazard ratio, *95% CI* 95% confidence interval.

**Table 3:** Cox univariate and multivariate analysis of predictors of recurrence in HCC patients following hepatectomy.

Variables for tumor recurrence	Univariate analysis	*p*-value	Multivariate analysis	*p*-value
	HR (95% CI)		HR (95%CI)	
Age, year (>50 versus ≤50)	1.513 (0.836–2.738)	0.171		
Gender (male versus female)	1.519 (0.471–4.896)	0.484		
HBsAg, (positive versus negative)	1.166 (0.544–2.501)	0.693		
Cirrhosis (present versus absent)	1.757 (0.545–5.666)	0.346		
Tumor encapsulation (complete/no)	1.474 (0.815–2.667)	0.200		
Tumor size, cm (>5 versus ≤5)	2.120 (1.177–3.820)	0.012	2.697 (1.433–5.077)	0.002
Tumor number (multiple versus single)	1.975 (0.919–4.242)	0.081	2.586 (1.133–5.906)	0.024
Edmondson–Steiner grade (I–II / III–IV)	1.618 (0.873–3.000)	0.126		
Vascular invasion (yes versus no)	1.612 (0.877–2.962)	0.124		
Preoperative AFP level, ng/ml (>400 versus ≤400)	0.961 (0.524–1.764)	0.899		
TNM stage (I/II–III)	1.606 (0.895–2.881)	0.112		
ACSL4 expression (high versus low)	1.641 (0.916–2.941)	0.096	1.663 (0.921–3.004)	0.092

Next, the expression level of ACSL4 was determined in several human HCC cell lines and normal human liver cell line QSG-7701. Consistent with the expression in tissue samples, the protein and mRNA expression level of ACSL4 was increased in almost all of the HCC cell lines using western blotting and qRT-PCR (Fig. 2e). These results indicate that ACSL4 expression is upregulated in HCC and is correlated with poor prognosis in HCC patients.

### ACSL4 promotes HCC cell proliferation in vitro

**Fig. 3:**
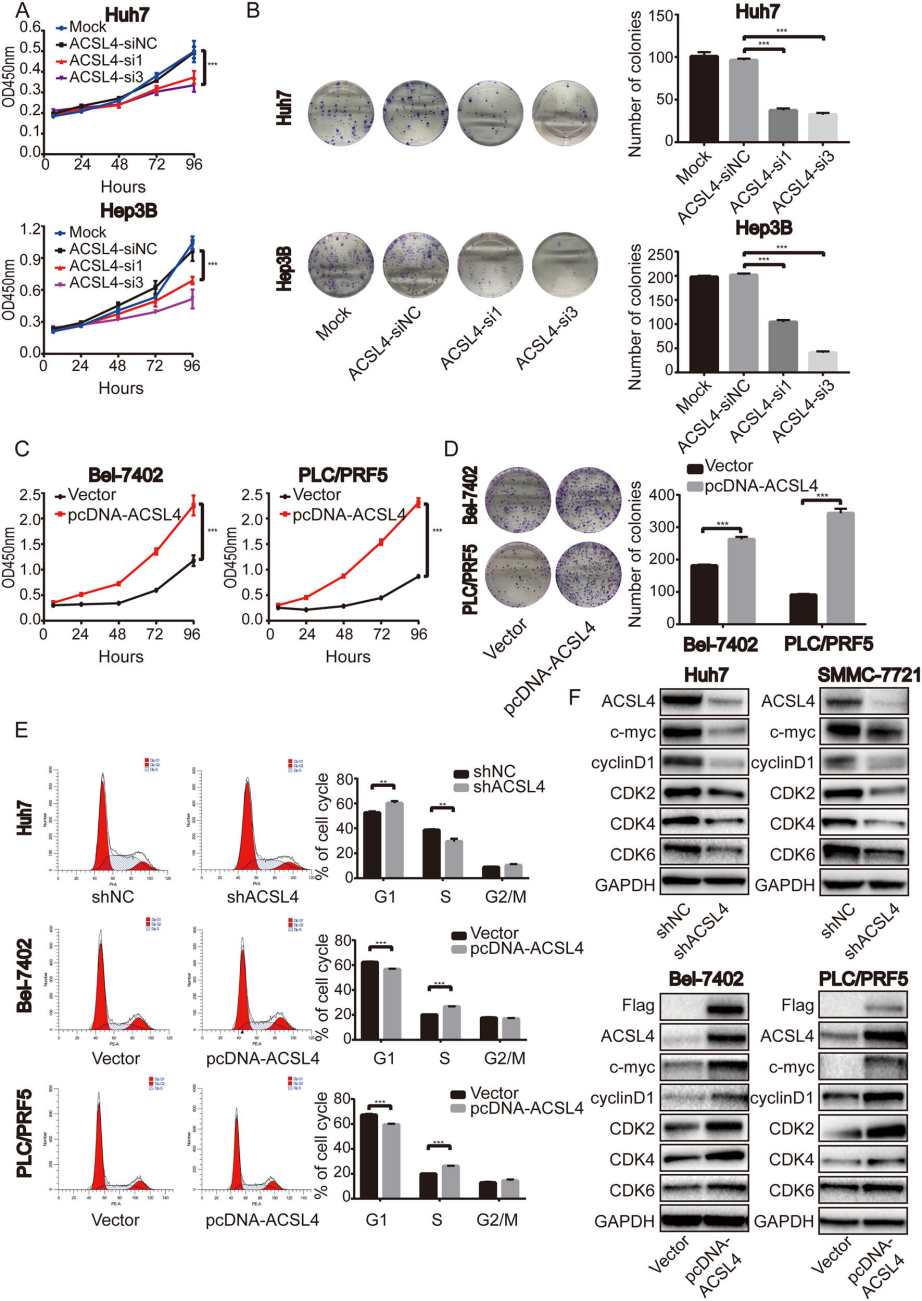
ACSL4 promotes the proliferation of HCC cells in vitro. **a** Effect of ACSL4 depletion on the proliferation of Huh7 and Hep3B cells by CCK-8 assay. **b** Photos for colony formation (left) and bar graph (right) in ACSL4-depleted Huh7 and Hep3B cells. **c** Effect of ACSL4 overexpression on the proliferation of Bel-7402 and PLC/PRF5 cells by CCK-8 assay. **d** Photos for colony formation (left) and bar graph (right) in ACSL4 overexpressed Bel-7402 and PLC/PRF5 cells. **e** Effect of ACSL4 depletion or overexpression on cell-cycle distribution in HCC cells by FACS. **f** Effect of ACSL4 depletion or overexpression on G1/S cell-cycle genes in HCC cells by western blotting. GAPDH was used as a loading control. Data are from three independent experiments and expressed as mean ± SD. **p < 0.01, ***p < 0.001. The data were analyzed using Student’s *t*-test.

According to the expression level of ACSL4 in HCC cell lines (Fig. 2e), high ACSL4-expressing HCC cell lines were selected to knockdown the expression level of ACSL4, whereas low ACSL4-expressing HCC cell lines were chosen to overexpress ACSL4. The knockdown or overexpression efficiency were confirmed through comparison with negative control at mRNA and protein levels (Supplementary Fig. S1). CCK-8 assays indicated that ACSL4 overexpression or knockdown significantly promoted or inhibited cell growth in corresponding HCC cells respectively (Fig. 3a, c). Furthermore, 2-dimension colony-formation assays showed that ACSL4 overexpression or knockdown significantly enhanced or impaired the colony-formation ability in corresponding HCC cells respectively (Fig. 3b, d). Consistent with these results, 5-ethynyl-2′-deoxyuridine (EdU) assays showed that HCC cell proliferation was impaired in ACSL4 knockdown group than those in control group (Supplementary Fig. S2).

It was reported that inhibition of ACSL4 could induce apoptosis in HCC cells^16^. Consistent with the previous studies, ACSL4 knockdown induced apoptosis and increased pro-apoptotic protein such as Bax and cleaved forms of caspase-3, whereas the anti-apoptotic protein Bcl-2 was downregulated in our context (Supplementary Fig. S3a, c). Moreover, Triacsin C, a specific inhibitor of ACSL4, recapitulating the consequences of ACSL4 knockdown (Supplementary Fig. S3b, d).

Cell cycle is another critical contributor to dysregulated cell growth and colony-formation ability. We asked whether ACSL4 could promote cell growth through cell cycle. We quantified cell-cycle distribution using flow cytometry and found ACSL4 knockdown in Huh7 cells induced G1 arrest (Fig. 3e). In contrast, Bel-7402 and PLC/PRF cells overexpressing ACSL4 showed an increase in S-phase cell population, with a concomitant decrease in cells in the G1 phase (Fig. 3e), indicating that ACSL4 accelerated G1/S progression. Consistent with these observations, depletion ACSL4 reduced G1/S cell-cycle protein expression of c-Myc, cyclinD1, CDK2, CDK4 and CDK6, and vice versa (Fig. 3f).

Collectively, these in vitro results suggest that ACSL4 promotes cell growth by activating cell-cycle progression and inhibiting apoptosis.

### ACSL4 enhances c-Myc stability at a post-transcription level

**Fig. 4:**
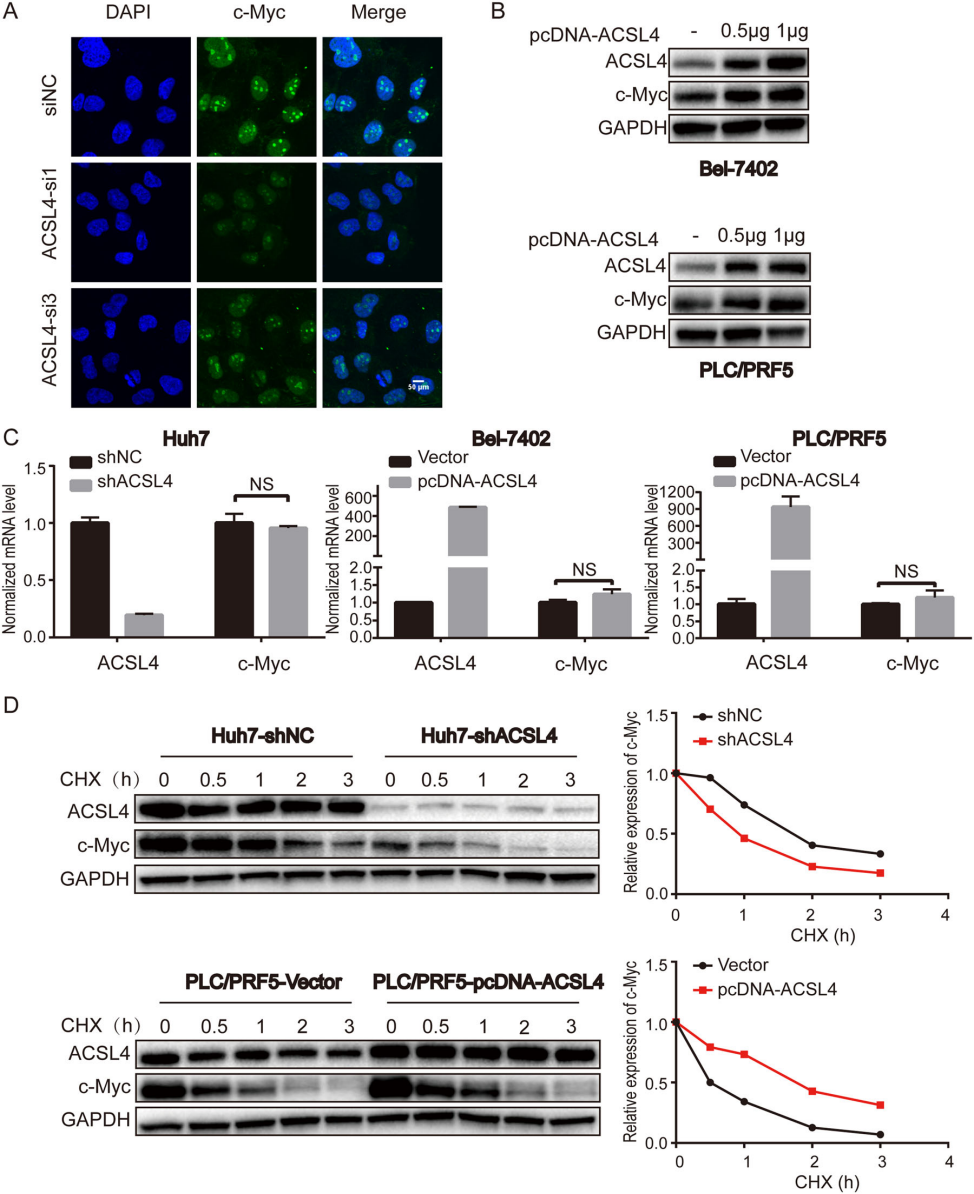
ACSL4 enhances c-Myc stability at a post-transcription level. **a** Immunofluorescence was conducted to visualize the expression of c-Myc in Huh7 cells with ACSL4 knockdown (c-Myc in green; DAPI in blue; scale bar: 50 μm). **b** Endogenous c-Myc protein levels following transfection (24 h) of 500 and 1000 ng of plasmid expressing pcDNA-ACSL4 in Bel-7402 and PLC/PRF5 cells. **c** qRT-PCR analysis of c-Myc in ACSL4 knockdown or overexpressed HCC cell lines. GAPDH was selected as the internal control. Data are presented as the mean ± SD (*n* = 3 biological replicates). The data were analyzed using Student’s *t*-test. NS: not significant. **d** Effect of protein synthesis inhibitor cycloheximide (CHX, 10 μg/ml) on c-Myc stability in ACSL4-depleted Huh7 cells and ACSL4 overexpressed PLC/PRF5 cells in a time course. The protein expression of ACSL4 and c-Myc was analyzed by western blotting (left) and semi-quantification (right). GAPDH was used as an internal standard.

The oncoprotein c-Myc affects cellular proliferation, survival pathways, and is frequently dysregulated in human tumors, including HCC. c-Myc functions as an essential regulator of G1/S transition by promoting the transcription of mRNAs encoding proteins that drive cell-cycle progression and cell growth^17^. So, we speculated that ACSL4 might promote HCC cell growth via c-Myc. Specifically, ACSL4 depletion significantly reduced the expression of c-Myc in HCC cells at protein level (Fig. 3f), which was supported by immunofluorescence (Fig. 4a). Furthermore, following the increase in ACSL4 expression, c-Myc expression exhibited an increasing trend in Bel-7402 and PLC/PRF5 cells (Fig. 4b). We next asked whether ACSL4 affects the expression of c-Myc at the transcriptional or post-transcription level. To answer this question, we used qRT-PCR to detect the mRNA level of c-Myc. Surprisingly, no significant difference was observed in the mRNA levels when ACSL4 overexpression or knockdown (Fig. 4c). Meanwhile, gene expression data from The Cancer Genome Atlas (TCGA) indicated that the lack of correlation between the mRNA levels of ACSL4 and c-Myc (data not shown). These results indicated that ACSL4 might affect c-Myc via a post-translational mechanism. Next, HCC cells were treated with de novo protein synthesis inhibitor, cycloheximide (CHX), at several specific time points. As expected, pretreatment with CHX resulted in a greater degradation c-Myc in the ACSL4 knockdown Huh7 cells, whereas the ectopic expression of ACSL4 in PLC/PRF5 cells led to a prolonged half-life of c-Myc (Fig. 4d), further supporting the notion that ACSL4 could stabilize c-Myc protein.

### ACSL4 regulates c-Myc through ubiquitin–proteasome system

**Fig. 5:**
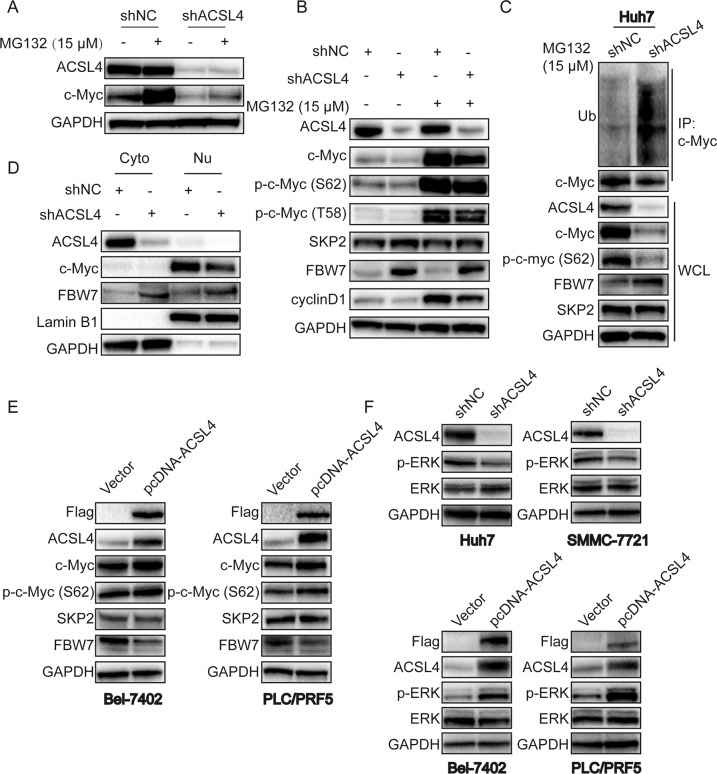
ACSL4 regulates c-Myc through ubiquitin–proteasome system. **a** Effect of proteosomal inhibitor MG132 on c-Myc expression in ACSL4-depleted Huh7 cells. Huh7-shNC and Huh7-shACSL4 cells were exposed to MG132 (15 μM) for 6–8 h for western blotting. **b** Effect of MG132 on c-Myc, p-c-Myc (T58), p-c-Myc (S62), SKP2, FBW7 and cyclinD1 in ACSL4-depleted Huh7 cells by western blotting. Huh7-shNC and Huh7-shACSL4 cells were exposed to MG132 (15 μM) for 6–8 h for western blotting. **c** Immunoprecipitation (IP) was used for the detection of c-Myc degradation in Huh7-shNC and Huh7-shACSL4 cells. After treating indicated cells with MG132 (15 μM) for 6–8 h, extracts were subjected to IP with c-Myc antibody and the polyubiquitination of c-Myc was assessed by western blot using ubiquitin antibody. Whole-cell lysates (WCL) were also subjected to western blot to detect indicated proteins. GAPDH protein was used as a loading control. **d** Western blotting of cytoplasmic and nuclear subcellular fractions from Huh7-shNC and Huh7-shACSL4 cells. GAPDH and Lamin B1 were used as internal standards. Cyto cytoplasm. Nu nucleus. **e** Effect of ACSL4 overexpression on c-Myc, p-c-Myc (S62), SKP2, FBW7 in Bel-7402 and PLC/PRF5 cells by western blotting. **f** Effect of ACSL4 depletion or overexpression on p-ERK in HCC cells by western blotting. GAPDH was used as a loading control.

c-Myc is a short-lived protein and a classic oncogene regulated at multiple steps. One of the most prominent mechanisms for c-Myc degradation in cells is through the ubiquitin–proteasome pathway^18^. We investigated the effect of proteasome inhibitor MG132 on c-Myc expression in ACSL4-depleted Huh7 cells. As expected, MG132 significantly reversed downregulation of c-Myc in Huh7-shACSL4 cells (Fig. 5a), suggested that the regulation may be related to the ubiquitination process. Phosphorylation of c-Myc at Thr58 or Ser62 has been shown to be important for the ubiquitin-dependent degradation of c-Myc, and S62 phosphorylation is necessary for the stability of the c-Myc protein and its activity^19,20^. In addition, phosphorylation of c-Myc (S62) and c-Myc (T58) were also reversed by treatment with MG132 (Fig. 5b). To date, c-Myc has have been reported to be degraded by several E3 ubiquitin ligases, such as FBW7 and SKP2. Interestingly, ACSL4 depletion increased the expression of FBW7, whereas SKP2 remained unchanged (Fig. 5b). Furthermore, ACSL4 depletion enhanced the ubiquitination of c-Myc (Fig. 5c). Nuclear fraction assay also revealed that ACSL4 depletion attenuated the expression of c-Myc in nucleus of Huh7 cells, along with increasing expression of FBW7 in cytoplasm and nucleus (Fig. 5d). In contrast, ectopic expression of ACSL4 enhanced c-Myc and phosphorylation of c-Myc (S62) expression, along with decreasing expression of FBW7 but not SKP2 in Bel-7402 and PLC/PRF5 cells (Fig. 5e).

Phosphorylation of c-Myc (S62) is known mediated by the MAPK/ERK pathway and contributes to the stabilization of c-Myc^21^. In addition, FBW7 has been reported to be phosphorylated by ERK kinase, which promotes FBW7 ubiquitination and destruction^22^. We hypothesized that ACSL4 may regulate the expression of c-Myc and FBW7 through ERK pathway. As anticipated, ACSL4 depletion effectively decreased phosphorylation of ERK in Huh7 and SMMC-7721 cells. Conversely, ACSL4 overexpression effectively increased phosphorylation of ERK in Bel-7402 and PLC/PRF5 cells (Fig. 5f). In addition, pretreatment Huh7 cells with ERK inhibitor SCH772984 markedly decreased c-Myc expression and increased FBW7 expression in a dose-dependent manner (Supplementary Fig. S4).

### c-Myc is the downstream effector of ACSL4-mediated HCC progression

**Fig. 6:**
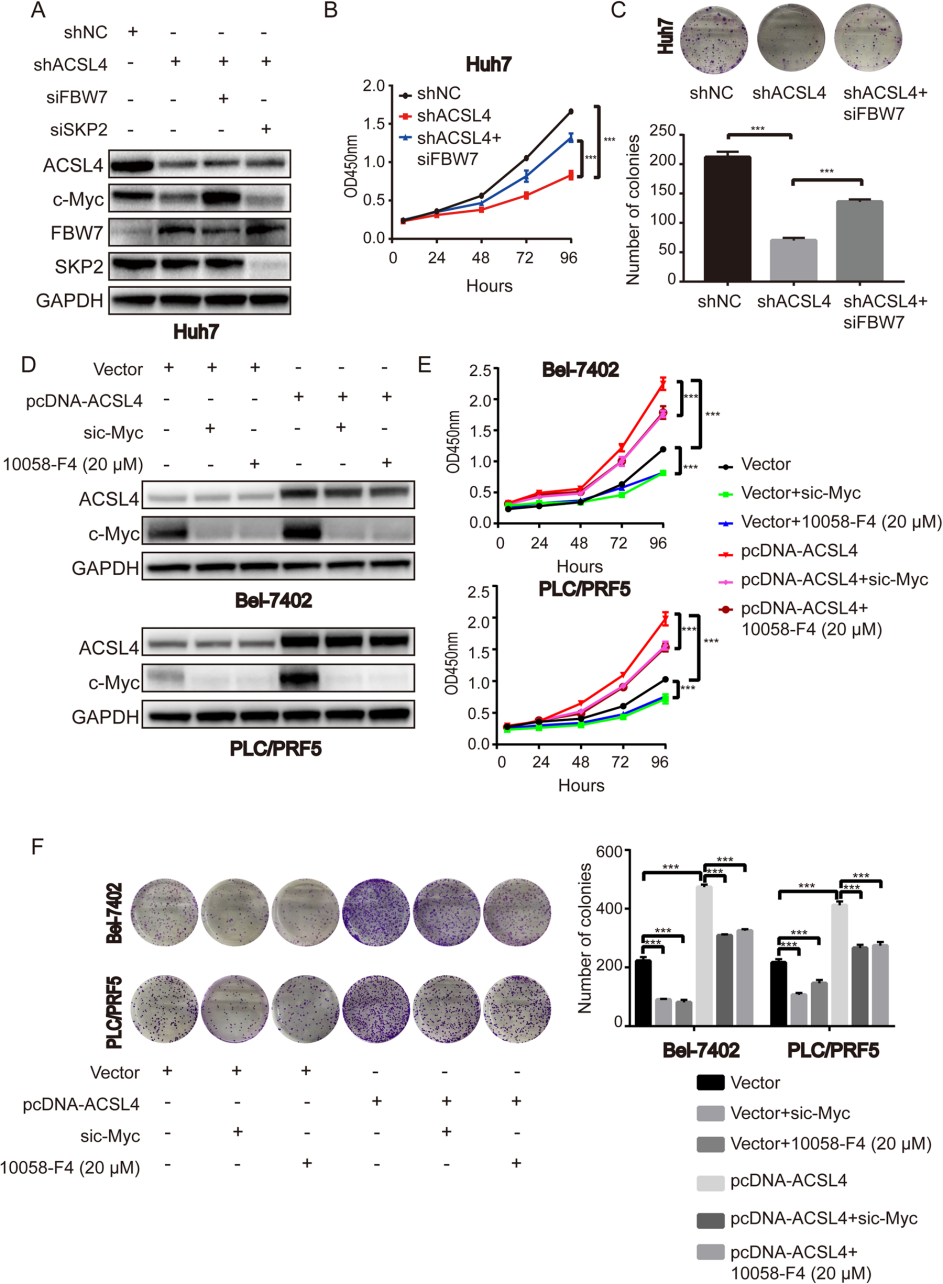
c-Myc is the downstream effector of ACSL4-mediated HCC progression. **a** Western blotting showed the expression levels of ACSL4, c-Myc, FBW7, SKP2 after treatment Huh7-shACSL4 cells with FBW7 or SKP2 siRNA. **b**, **c** CCK-8 and colony-formation assays for indicated Huh7 cells were evaluated. **d** Western blotting showed the suppression efficiency after treatment Bel-7402-pcDNA-ACSL4 and PLC/PRF5-pcDNA-ACSL4 cells with c-Myc siRNA or a specific c-Myc inhibitor 10058-F4 (20 μM). **e**, **f** CCK-8 and colony-formation assays for indicated Bel-7402 and PLC/PRF5 cells were evaluated. Data are presented as the mean ± SD (*n* = 3 biological replicates). ***p < 0.001. The data were analyzed using Student’s *t*-test.

As c-Myc is an important oncoprotein, we next investigated whether c-Myc participates in the ACSL4-mediated HCC cell proliferation. Western blotting was performed after treatment Huh7-shACSL4 cells with FBW7 or SKP2 siRNA. FBW7 siRNA remarkably restored c-Myc protein level mediated by ACSL4 depletion (Fig. 6a). However, SKP2 siRNA treatment had no obvious effect on c-Myc protein in Huh7 cells (Fig. 6a). CCK-8 and colony-formation assays demonstrated that siFBW7 partly restored the suppressed proliferation in Huh7-shACSL4 cells (Fig. 6b, c). Also, we employed both c-Myc siRNAs and inhibitors to achieve c-Myc inhibition. Treatment Bel-7402-pcDNA-ACSL4 and PLC/PRF5-pcDNA-ACSL4 cells with c-Myc siRNA effectively inhibited the expression of c-Myc, but ACSL4 protein level remained unchanged (Fig. 6d). Accordingly, treatment of a specific c-Myc inhibitor, 10058-F4, resulted in similar results (Fig. 6d). CCK-8 and colony-formation assays showed that inhibition of c-Myc by siRNA or its inhibitor partly rescued the promoted proliferation in ACSL4 overexpressing HCC cells (Fig. 6e, f). Taken together, these findings indicate that c-Myc is the downstream effector of ACSL4-mediated HCC progression.

### ACSL4 depletion suppresses the growth of HCC cells and c-Myc protein level in vivo

**Fig. 7:**
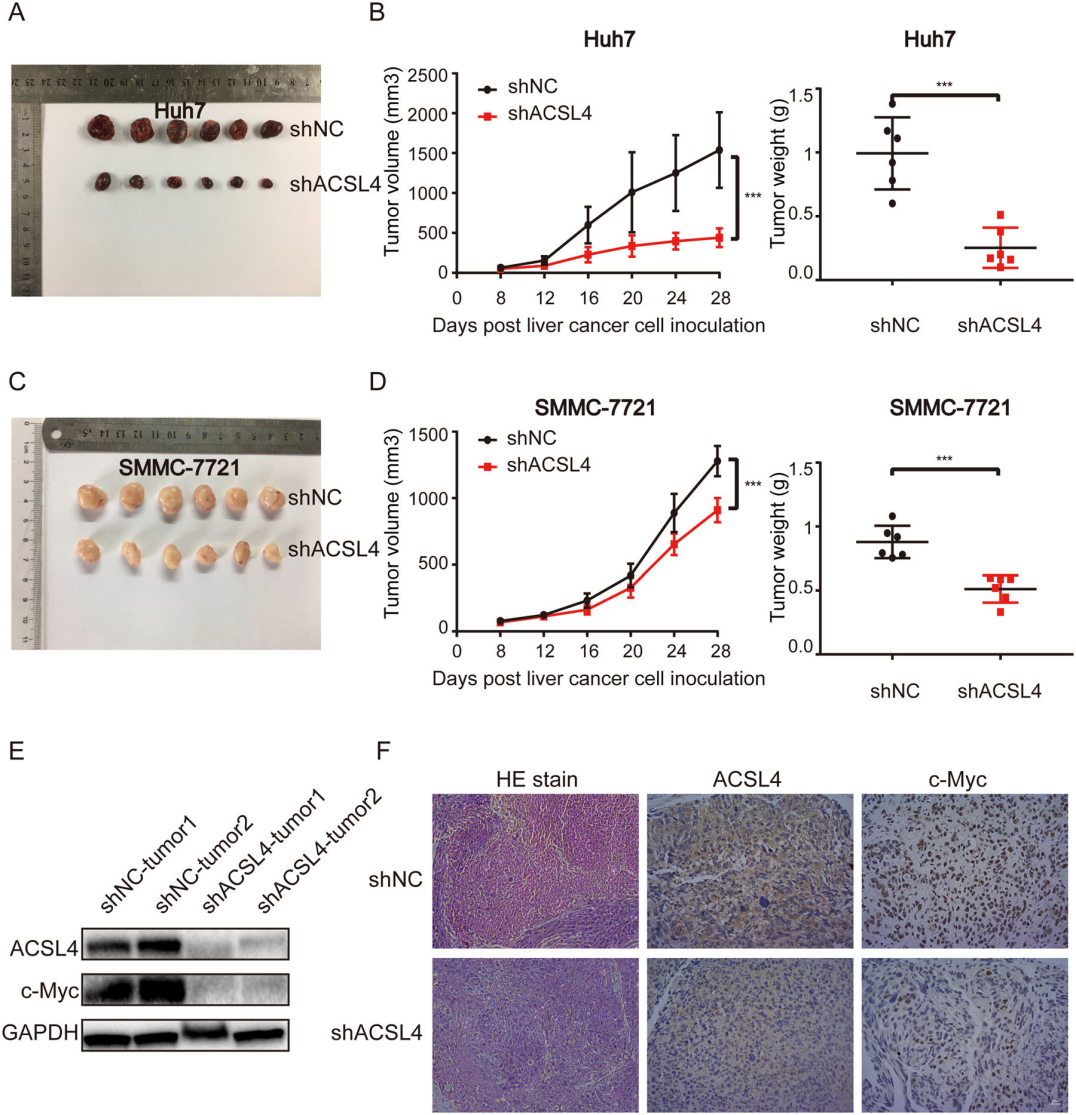
ACSL4 depletion suppresses the growth of HCC cells and c-Myc protein level in vivo. **a** Representative images of tumor size in nude mice injected subcutaneously with Huh7 cells infected with either shNC or shACSL4 (*n* = 6, for each experimental group). The mice were killed on the 28th day for tumor weight analysis. **b** Tumor growth rates and tumor weight in Huh7-shNC group and Huh7-shACSL4 group. **c** Representative images of tumor size in nude mice injected subcutaneously with SMMC-7721 cells infected with either shNC or shACSL4. The mice were killed on the 28th day for tumor weight analysis (*n* = 6, for each experimental group). **d** Tumor growth rates and tumor weight in SMMC-7721-shNC group and SMMC-7721-shACSL4 group. The data shown are the mean ± SD of tumor volume and tumor weight from six nude mice per group (***p < 0.001). The data were analyzed using Student’s *t*-test. **e** ACSL4 and c-Myc protein levels in Huh7 indicated xenografts were detected by western blot. **f** H&E staining and the expression of ACSL4 and c-Myc in tumor sections by IHC analysis (scale bar: 100 μm; magnification: ×200).

Because our in vitro studies suggested a functional role for ACSL4 in HCC proliferation and cell cycle, we investigated the contribution of ACSL4 to HCC growth in vivo. The effect of ACSL4 on primary tumor growth was evaluated in athymic nude mice bearing ACSL4 stably depleted Huh7 and SMMC-7721 cells. As shown in Fig. 7a, c, the tumor size was significantly smaller in shACSL4 group than in shNC group. Tumor volumes in ACSL4-depleted group were significantly reduced compared to the negative control group (*p* < 0.05) (Fig. 7b, d). Similarly, at necropsy, the excised tumor weights were significantly suppressed in ACSL4-depleted group compared to negative control (Fig. 7b, d) with decreased expression of c-Myc and ACSL4 (Fig. 7e, f). To summarize, these results indicate that ACSL4 promotes tumor growth in vivo, which is consistent with our in vitro and clinical findings.

### ACSL4 expression is positively correlated with c-Myc in HCC

**Fig. 8:**
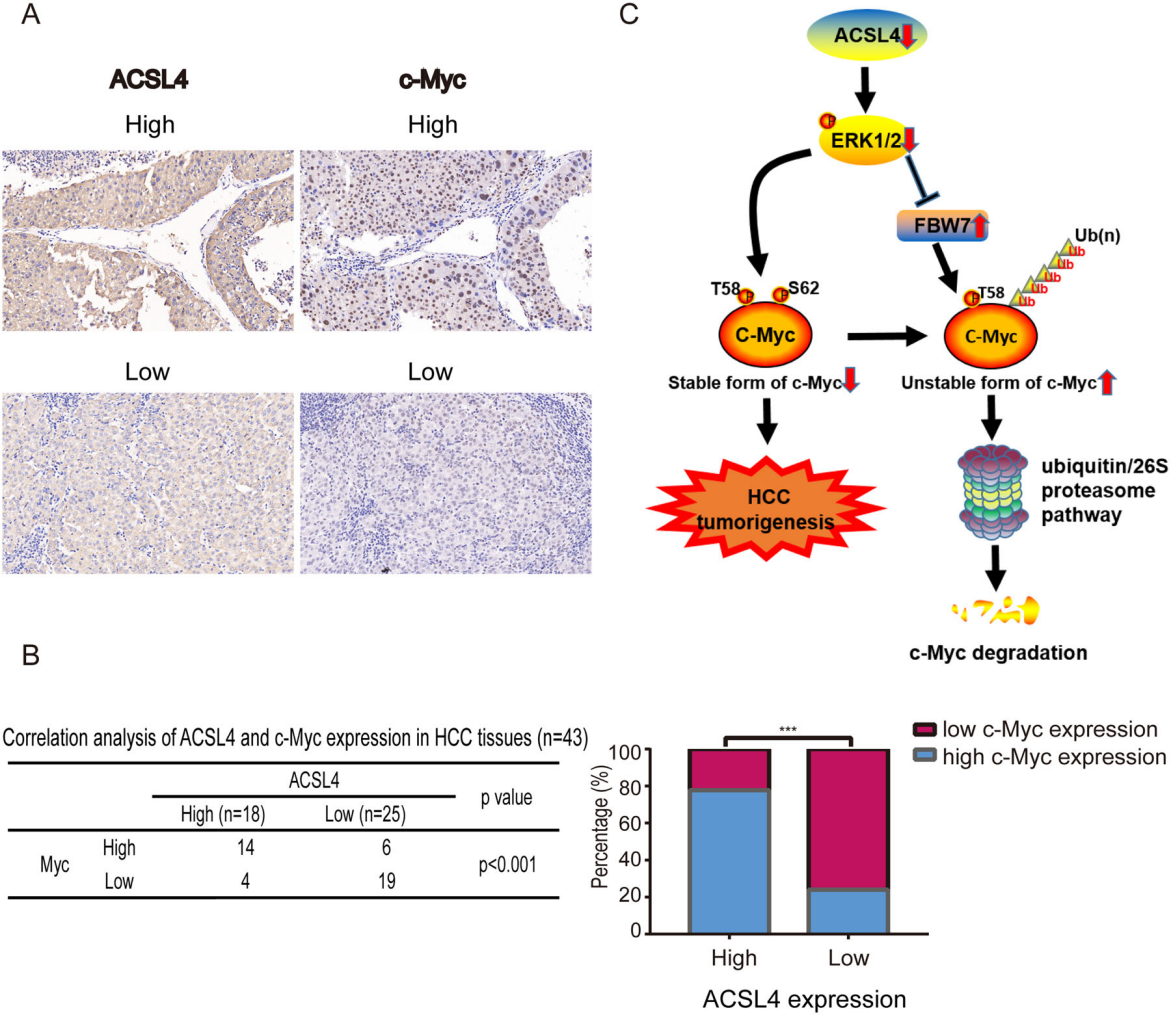
ACSL4 expression is positively correlated with c-Myc in HCC. **a** c-Myc expression was higher in patients with higher ACSL4 expression, as suggested by IHC staining (scale bar: 20 μm; magnification: ×400). **b** Correlation between ACSL4 levels and nuclear expression of c-Myc in an HCC array. The histogram (right) showed the percentages of tumors with low or high nuclear expression of c-Myc in those with low (*n* = 25) or high (*n* = 18) expression of ACSL4. ***p < 0.001. The correlations were assessed with χ^2^ test. **c** Our results suggest a model in which depletion of ACSL4 results in reduction of p-ERK, thus decreases phospho-S62 c-Myc and increases the expression of one of the c-Myc E3 ubiquitin ligases, FBW7. Dephosphorylation c-Myc at S62 in the context of phospho-T58 and FBW7 overexpression thereby results in the ubiquitin-mediated degradation of c-Myc. As such, ACSL4 depletion ultimately results in the inhibition of c-Myc oncogenic activity and suppressed hepatocellular carcinogenesis.

To further explore the correlation between ACSL4 and c-Myc, we performed IHC staining to examine the expression correlation between ACSL4 and c-Myc. HCC patients that displayed higher ACSL4 expression exhibited higher c-Myc expression (Fig. 8a). Approximately 77.8% of samples with high ACSL4 expression displayed strong staining of c-Myc, whereas 22.2% showed weak staining for c-Myc. Similarly, 76% of samples with low ACSl4 expression displayed weak expression of c-Myc, and 24% displayed high levels of c-Myc (Fig. 8b). These results indicated that the expression of ACSL4 and c-Myc were negatively correlated in clinical samples. Thus, ACSL4 could promote HCC progression through the ERK/FBW7/c-Myc axis (Fig. 8c).

## Discussion

HCC exhibits high heterogeneity. More evidence has revealed that HCC accompanied with high AFP concentrations have a distinct tumor biology and could be regarded as a subtype of the disease. Here, we identified ACSL4 as a novel marker for AFP^high^ HCC patients through transcriptomic array. Our results regarding the correlation of ACSL4 with AFP levels, providing the clinical evidence of the association of ACSL4 with HCC progression, although the significance of the analysis may be needed to be further investigated in more HCC cohorts.

ACSL4 has been demonstrated to have important roles in human malignant tumors, including breast cancer^11^, prostate cancer^14^, colon adenocarcinoma^12^ and gastric cancer^13^. However, roles of ACSL4 vary in different cancer types. Interestingly, recent studies uncovered ACSL4 as an essential component for ferroptosis execution^23,24^. However, the role of ACSL4 in HCC has not been systematically investigated and its underlying mechanism remains elusive.

The clinical significance, biological function, and molecular regulation of ACSL4 were investigated in this study. We demonstrated that ACSL4 was widely upregulated in HCC tissues and cell lines. Survival analyses indicated that ACSL4 overexpression predicted unfavorable OS and RFS in HCC patients. These results suggest that ACSL4 acts as an oncogene in HCC. In addition, we found that ACSL4 promotes HCC cell proliferation both in vivo and in vitro.

Subsequently, we attempted to investigate the potential biological function and molecular mechanism of ACSL4 in HCC progression. Notably, we demonstrated that ACSL4 depletion abrogated cell proliferation, induced G1 arrest, downregulated c-Myc and c-Myc-related cell-cycle proteins, indicating that ACSL4 promotes cell growth by activating cell-cycle progression.

MYC (c-Myc) is a very well-known oncogene and is a master transcription factor that regulates the expression of 10% to more than 15% of all cellular genes^25^, which has been identified as one of the most commonly deregulated oncogenes in a wide variety of cancer types^26,27^. It has been reported that the conditional inactivation of c-Myc can be sufficient to induce sustained regression of HCC^28^. Thus, targeted inactivation of c-Myc may be an effective therapy for HCC^29,30^. However, the exact mechanisms of c-myc activation in HCC are largely unknown. One of the main biological functions of c-Myc is to promote cell-cycle progression^31^, so we hypothesized that ACSL4 modulates the cell cycle in HCC by regulating c-Myc. Through our HCC tissue samples, we confirmed that ACSL4 and c-Myc expression were positively correlated. Our further study found that ACSL4 increased the expression of c-Myc protein, but had no effect on c-Myc mRNA in vitro and in vivo, suggesting that ACSL4 might regulate c-Myc expression at a post-transcriptional level. Proteasome-mediated degradation was very important for the turnover of many proteins, including c-Myc. We found that ACSL4 depletion promoted the degradation of c-Myc induced by ubiquitination, indicating proteasomal dependent c-Myc degradation by ACSL4 depletion. Two key phosphorylation sites of Ser62 and Thr58 located within the N terminus are important for the regulation of c-Myc protein stabilization, yet have opposite function. Ser62 phosphorylation stabilizes c-Myc protein, whereas T58 phosphorylation leads to its protein degradation^32^. We found that ACSL4 overexpression prolonged, while depletion attenuated, c-Myc half-lives after treatment with cycloheximide (CHX). However, proteasome inhibitor MG132 reversed the phosphorylation of c-Myc (S62) and c-Myc (T58), implying ACSL4 enhances c-Myc stability via blockade of c-Myc degradation by ubiquitin–proteasome system. In addition, c-Myc did not affect the expression of ACSL4 in our HCC cells (Fig. 6d), indicating ACSL4 is an upstream of c-Myc.

E3 ligase-mediated protein ubiquitination and degradation are the mechanisms responsible for most of protein degradation through a proteasome-dependent manner^33^. FBW7 and SKP2 are well-known E3 ligases, which have been mostly reported to be responsible for c-Myc degradation. In the present study, FBW7 but not SKP2 depletion restored the decreased expression of c-Myc in Huh7 cells, indicating inverse interaction between FBW7 and c-Myc in HCC. Notably, ACSL4 depletion increased the expression of FBW7, whereas SKP2 remained unchanged, and vice versa, implying that ACSL4 acts as an upstream of FBW7 and c-Myc. Moreover, the tumorigenic effects driven by ACSL4 could be partly reversed by c-Myc knockdown or its inhibitor 10058-F4, while the effects of ACSL4 silencing were partially rescued by c-Myc overexpression via FBW7 knockdown. In addition to the E3 ubiquitin ligases (FBW7, SKP2) in this study, other proteins can regulate the degradation process of c-Myc. It has been reported that deubiquitinase USP28 and c-Myc can form a positive feedback loop that maintains high c-Myc protein levels in tumors^34^. Another study found that the proteasome activator REGγ controls the abundance of c-Myc proteins by ubiquitination-independent pathway^35^. In our work, we investigated the most frequently E3 ligases, especially FBW7, whose expression is reduced and is inversely correlated with its substrate c-Myc in HCC tissues^36^. Whether other E3 ligases and deubiquitylases or whether one or more other mechanisms (namely, ubiquitination-independent pathway) may be involved in ACSL4-mediated c-Myc stability needs our future work. Also, the inhibitory effect caused by c-Myc disruption is not complete, suggesting that there may be other c-Myc-independent molecules involved in ACSL4-mediated oncogenic process. Taken together, these data strongly demonstrate that c-Myc is required for ACSL4 to promote HCC progression.

Several evidences showed that phosphorylation of c-Myc serine 62 (pS62) is a key phenomenon to stabilize c-Myc^37^, which is mediated by extracellular signal-regulated kinase (ERK) signaling^38^. ERK can also interact with and phosphorylate FBW7 at Thr205, leading to FBW7 to be unstable and regulated by proteasomal degradation through self-polyubiquitination^22^. Here, ACSL4 depletion decreased phosphorylation of ERK leading to downregulation of phosphorylation of c-Myc serine 62 (pS62) and upregulation of FBW7 in Huh7 cells, which was reversed by ERK specific inhibitor SCH772984, indicating that ERK signaling has a key role in ACSL4-mediated c-Myc stability in HCC. Aberrant activation of the RAS-ERK pathway is involved in the progression of human HCC and complex mechanisms lead to activation of the RAS pathway^39^. The pathway by which ACSL4 activates the ERK signaling (enzyme-dependent or enzyme-independent manner) requires our further study. To the best of our knowledge, this study is the first to reveal that ACSL4 could promote HCC progression through the ERK/FBW7/c-Myc axis (Fig. 8c). Thus, our study identified that ACSL4 had an important role in the development and progression of HCC and also advanced the understanding of mechanisms regulating c-Myc stability.

In summary, our study identified ACSL4 as a novel marker for AFP high subtype HCC and revealed a novel mechanism by which ACSL4 acts as an oncogene in the progression of HCC by mediating the ubiquitination and degradation of c-Myc. Considering c-Myc protein is un-druggable and lack obvious pockets where small molecules or drugs can bind^40^, interruption of c-Myc regulatory units like ACSL4 might be a promising alternative to indirectly target c-Myc protein in HCC. These findings suggest that ACSL4 could be a valuable prognostic biomarker and a potential therapeutic target in HCC.

## Supplementary information

Supplementary Table 1

Supplementary Table 2

Supplementary Table 3

Supplementary figure legends

Supplementary figure S1

Supplementary figure S2

Supplementary figure S3

Supplementary figure S4
