# Supplementary material for: ACSL4 promotes hepatocellular carcinoma progression via c-Myc stability mediated by ERK/FBW7/c-Myc axis
Source: Oncogenesis. 2020 Apr 29;9(4):42. doi: 10.1038/s41389-020-0226-z (PMC7190855; doi:10.1038/s41389-020-0226-z)
Supplement: Supplementary file 1 — Supplementary Table 1 [file 41389_2020_226_MOESM1_ESM.doc]

**Supplementary Table 1. Clinical features of 6 HCC patients for transcriptomic array.**

| Features | AFP^low^ group (n=3) | AFP^high^ group (n=3) |
| --- | --- | --- |
| Age (years) | Median 50, range 49-55 | Median 50, range 45-63 |
| Gender | Male 3 (100%) | Male 3 (100%) |
| HBsAg positive (yes/non) | Yes 3 (100%) | Yes 3 (100%) |
| Liver cirrhosis (yes/non) | Yes 3 (100%) | Yes 3 (100%) |
| Tumor diameter (cm, mean ± SD) | 5.10± 0.98 | 9.03±2.65 |
| Tumor number (1/2/3) | 3 (100%)/0/0 | 1 (33%)/0/2 (67%) |
| Venous invasion (yes/no) | No 3 (100%) | Yes 3 (100%) |
| Alpha-fetoprotein (ng/mL) | Median 3, range 2-8.1 | Median 29277, range 12806-30748 |
