# Supplementary material for: ACSL4 promotes hepatocellular carcinoma progression via c-Myc stability mediated by ERK/FBW7/c-Myc axis
Source: Oncogenesis. 2020 Apr 29;9(4):42. doi: 10.1038/s41389-020-0226-z (PMC7190855; doi:10.1038/s41389-020-0226-z)
Supplement: Supplementary file 2 — Supplementary Table 2 [file 41389_2020_226_MOESM2_ESM.doc]

**Supplementary Table 2. TOP 57 upregulated transcripts in AFP^high^ group and HCC tissues**

| Probe Set ID | Gene | Fullname of Gene |
| --- | --- | --- |
| TC01000382.hg.1 | RNU105A | RNA, U105A small nucleolar |
| TC11003382.hg.1 | S59749 | #N/A |
| TC05001408.hg.1 | CENPK | centromere protein K |
| TC17000355.hg.1 | ATAD5 | ATPase family, AAA domain containing 5 |
| TC04000155.hg.1 | NCAPG | non-SMC condensin I complex, subunit G |
| TC10000659.hg.1 | KIF11 | kinesin family member 11 |
| TC01003889.hg.1 | ENAH | enabled homolog (Drosophila) |
| TC02002376.hg.1 | MCM6 | minichromosome maintenance complex component 6 |
| TC06001799.hg.1 | MCM3 | minichromosome maintenance complex component 3 |
| TC12000974.hg.1 | KNTC1 | kinetochore associated 1 |
| TC10002227.hg.1 | AK021443 | #N/A |
| TC10000673.hg.1 | HELLS | helicase, lymphoid-specific |
| TC10002226.hg.1 | BC105606 | #N/A |
| TC20000637.hg.1 | ESF1 | ESF1, nucleolar pre-rRNA processing protein, homolog (S. cerevisiae) |
| TC02000615.hg.1 | C2orf29 | chromosome 2 open reading frame 29 |
| TC02003441.hg.1 | CR614528 | #N/A |
| TC08001736.hg.1 | EPPK1 | epiplakin 1 |
| TC15000701.hg.1 | DQ588677 | #N/A |
|  |  |  |
| TC20000822.hg.1 | RBL1 | retinoblastoma-like 1 (p107) |
| TC17002366.hg.1 | NR_038131 | #N/A |
| TC0X000385.hg.1 | NONO | non-POU domain containing, octamer-binding |
| TC0X000323.hg.1 | MAGED2 | melanoma antigen family D, 2 |
| TC0X002337.hg.1 | **ACSL4** | acyl-CoA synthetase long-chain family member 4 |
| TC15000868.hg.1 | BLM | Bloom syndrome, RecQ helicase-like |
| TC20000784.hg.1 | PIGU | phosphatidylinositol glycan anchor biosynthesis, class U |
| TC06000668.hg.1 | LRRC1 | leucine rich repeat containing 1 |
| TC09002860.hg.1 | NR_024542 | #N/A |
| TC12001701.hg.1 | cdna:known chromosome:GRCh37:12:68946775:68947662:-1 gene:ENSG00000240087 gene_biotype:pseudogene transcript_biotype:retrotransposed | #N/A |
| TC0X002285.hg.1 | NR_024450 | #N/A |
| TC16001511.hg.1 | linc-TP53TG3B-18 chr16:+:32881354-32884886 | #N/A |
| TC16001863.hg.1 | linc-TP53TG3B-6 chr16:-:33790305-33794149 | #N/A |
| TC20000384.hg.1 | CSE1L | CSE1 chromosome segregation 1-like (yeast) |
| TC10000644.hg.1 | KIF20B | kinesin family member 20B |
| TC10002200.hg.1 | BC005221 | #N/A |
| TC10000098.hg.1 | MCM10 | minichromosome maintenance complex component 10 |
| TC17000495.hg.1 | CDC6 | cell division cycle 6 homolog (S. cerevisiae) |
| TC03001632.hg.1 | KIAA1524 | KIAA1524 |
| TC20000198.hg.1 | TPX2 | TPX2, microtubule-associated, homolog (Xenopus laevis) |
|  |  |  |
| TC04000477.hg.1 | HSP90AB3P | heat shock protein 90kDa alpha (cytosolic), class B member 3, pseudogene |
| TC01005497.hg.1 | NR_033142 | #N/A |
| TC15000449.hg.1 | CCNB2 | cyclin B2 |
| TC04001419.hg.1 | H2AFZ | H2A histone family, member Z |
| TC05000298.hg.1 | CCNB1 | cyclin B1 |
| TC10000373.hg.1 | CDK1 | cyclin-dependent kinase 1 |
| TC20000592.hg.1 | PCNA | proliferating cell nuclear antigen |
| TC08001481.hg.1 | PABPC1 | poly(A) binding protein, cytoplasmic 1 |
| TC12000779.hg.1 | GAS2L3 | growth arrest-specific 2 like 3 |
| TC08000412.hg.1 | CHD7 | chromodomain helicase DNA binding protein 7 |
| TC08001940.hg.1 | AB037837 | #N/A |
| TC15002602.hg.1 | linc-TLE3-2 chr15:-:70588400-70590244 | #N/A |
| TC20000618.hg.1 | ncrna:miRNA chromosome:GRCh37:20:10342736:10342878:-1 gene:ENSG00000211588 gene_biotype:miRNA transcript_biotype:miRNA | #N/A |
| TC01003452.hg.1 | OLFML2B | olfactomedin-like 2B |
| TC06002025.hg.1 | LAMA4 | laminin, alpha 4 |
| TC06002737.hg.1 | BC070230 | #N/A |
| TC06001751.hg.1 | XPO5 | exportin 5 |
| TC02003327.hg.1 | NR_029403 | #N/A |
|  |  |  |
| TC17002860.hg.1 | KPNA2 | karyopherin alpha 2 (RAG cohort 1, importin alpha 1) |
|  |  |  |
