# Supplementary material for: ACSL4 promotes hepatocellular carcinoma progression via c-Myc stability mediated by ERK/FBW7/c-Myc axis
Source: Oncogenesis. 2020 Apr 29;9(4):42. doi: 10.1038/s41389-020-0226-z (PMC7190855; doi:10.1038/s41389-020-0226-z)
Supplement: Supplementary file 3 — Supplementary Table 3 [file 41389_2020_226_MOESM3_ESM.doc]

**Supplementary Table 3. Clinicopathological features of 87 HCC patients enrolled in this study (Cohort2).**

| Features | Values/counts |
| --- | --- |
| Age (years) | Median 52, range 31-78 |
| Gender | (Male/female) 79 (90.8%)/8 (9.2%) |
| HBsAg positive (yes/non) | 70 (80.5%)/17 (19.5%) |
| Liver cirrhosis (yes/non) | 78 (89.7%)/9 (10.3%) |
| Tumor encapsulation (complete/no) | 40 (46%)/47 (54%) |
| Tumor diameter (cm, mean ± SD) | 4.79 ± 3.11 |
| Tumor number (1/2/3) | 77 (88.5%)/9 (10.3%)/1 (1.1%) |
| Edmondson-Steiner grade (I-II/III-IV) | 64 (73.6%)/23 (26.4%) |
| Venous invasion (yes/no) | 24 (27.6%)/ 63 (72.4%) |
| Alpha-fetoprotein (ng/mL) | Median 73, range 1-60500 |
| TNM stage (I/II/IIIA) | 57 (65.5%)/28 (32.2%)/2 (2.3%) |
