# Supplementary material for: ACSL4 promotes hepatocellular carcinoma progression via c-Myc stability mediated by ERK/FBW7/c-Myc axis
Source: Oncogenesis. 2020 Apr 29;9(4):42. doi: 10.1038/s41389-020-0226-z (PMC7190855; doi:10.1038/s41389-020-0226-z)
Supplement: Supplementary file 4 — Supplementary figure legends [file 41389_2020_226_MOESM4_ESM.doc]

**Fig.S1.** Validation of ACSL4 knockdown or overexpression in HCC cell lines.

(A) The BLOCK-iT™ Alexa Fluor™ Red Fluorescent Control (Thermo Scientific, USA) was transfected into Huh7 and Hep3B cells to validate the transfection efficiency. Twenty-four hours after the start of transfection, the oligo was seen with transfection reagents. Almost 90% of the cells take up the oligo, and cells retain a normal morphology, as seen in the bright-field images (scale bar: 200 μm; magnification: 100×). (B) ACSL4 knockdown efficiency in Huh7 and Hep3B cells was confirmed by qRT-PCR and Western blot analysis. (C) Overexpression of ACSL4 in Bel-7402 and PLC/PRF5 cells was confirmed by qRT-PCR and Western blot analysis.

**Fig.S2.** ACSL4 depletion impairs cell proliferation in HCC cell lines.

EdU assays were used to evaluate the effect of ACSL4 knockdown on DNA synthesis in Huh7 and Hep3B cells. Representative images and corresponding statistical plots were presented (scale bar: 200 μm; magnification: 200×). *P < 0.05, **P < 0.01, ***P < 0.001. The data were analyzed using Student’s t-test.

**Fig.S3.** ACSL4 depletion or inhibition induces apoptosis in HCC cell lines.

(A) ACSL4 knockdown in Huh7 and Hep3B cells induced apoptosis, as determined by flow cytometry. (B) ACSL4 inhibition by triacsin C treatment (10 μM, 20 μM) for 24 h induced apoptosis, which could be reversed by a pan-caspase inhibitor, Z-VAD-FMK (50 μM). DMSO was used as control. Data were represented as mean ± SD of at least three independent experiments. All **P < 0.01, ***P < 0.001. The data were analyzed using Student’s t-test. (C) Effect of ACSL4 depletion on cell apoptosis-related genes in Huh7 and Hep3B cells by western blotting. GAPDH was used as a loading control. (D) Effect of ACSL4 inhibition by triacsin C treatment on cell apoptosis-related genes in Huh7 cells by western blotting. GAPDH was used as a loading control.

**Fig.S4.** ERK inhibition decreases c-Myc expression and increases FBW7 expression in Huh7 cells.

Huh7 cells were exposed to ERK inhibitor SCH772984 (5 μM, 10 μM) for 24 h. Western blotting showed the expression levels of p-ERK, ERK, c-Myc, p-c-Myc (S62) and FBW7. GAPDH was used as a loading control.
