# Supplementary figures and images for: ACSL4 promotes hepatocellular carcinoma progression via c-Myc stability mediated by ERK/FBW7/c-Myc axis
Source: Oncogenesis. 2020 Apr 29;9(4):42. doi: 10.1038/s41389-020-0226-z (PMC7190855; doi:10.1038/s41389-020-0226-z)

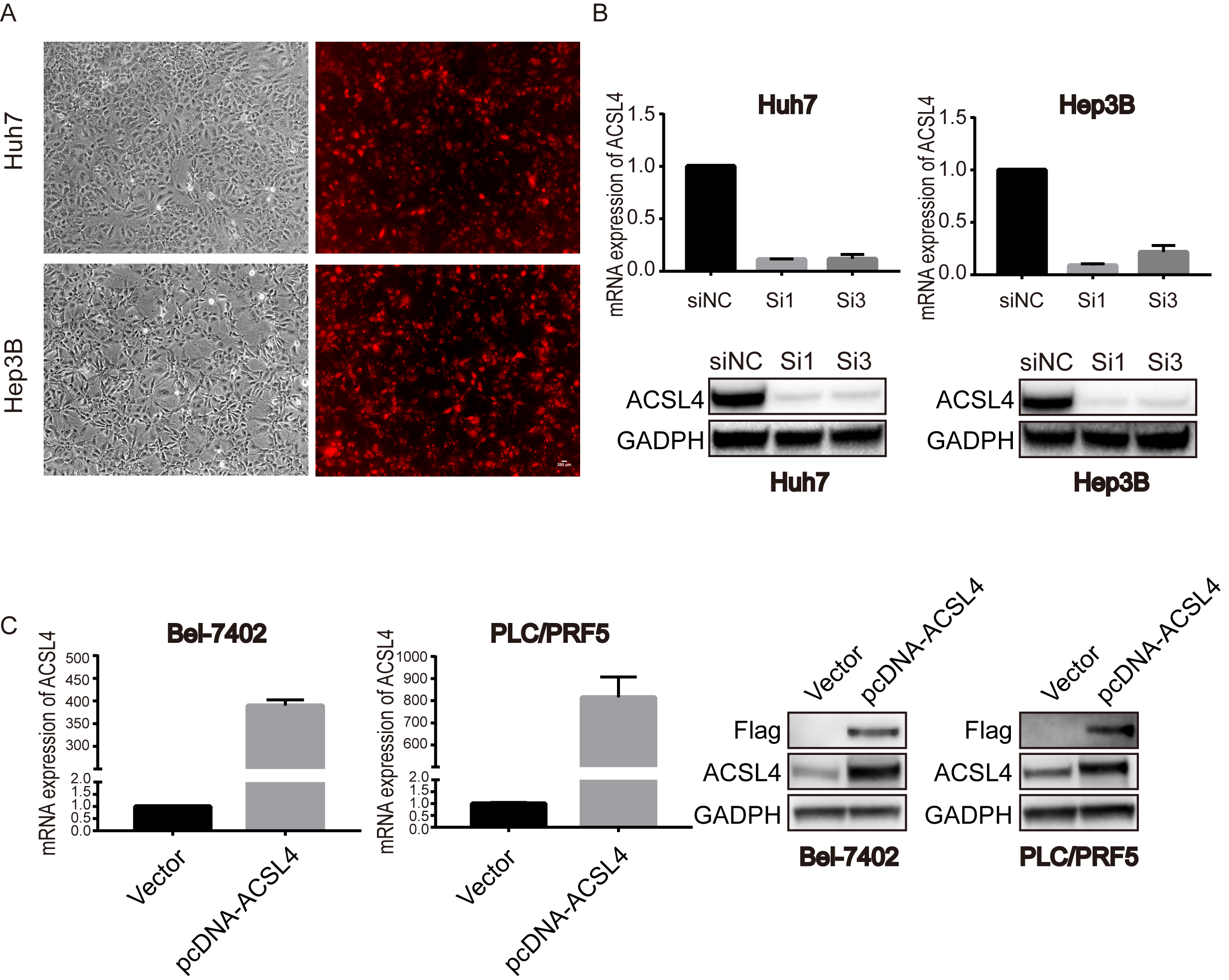

Supplement: Supplementary file 5 — Supplementary figure S1 [file 41389_2020_226_MOESM5_ESM.tif]

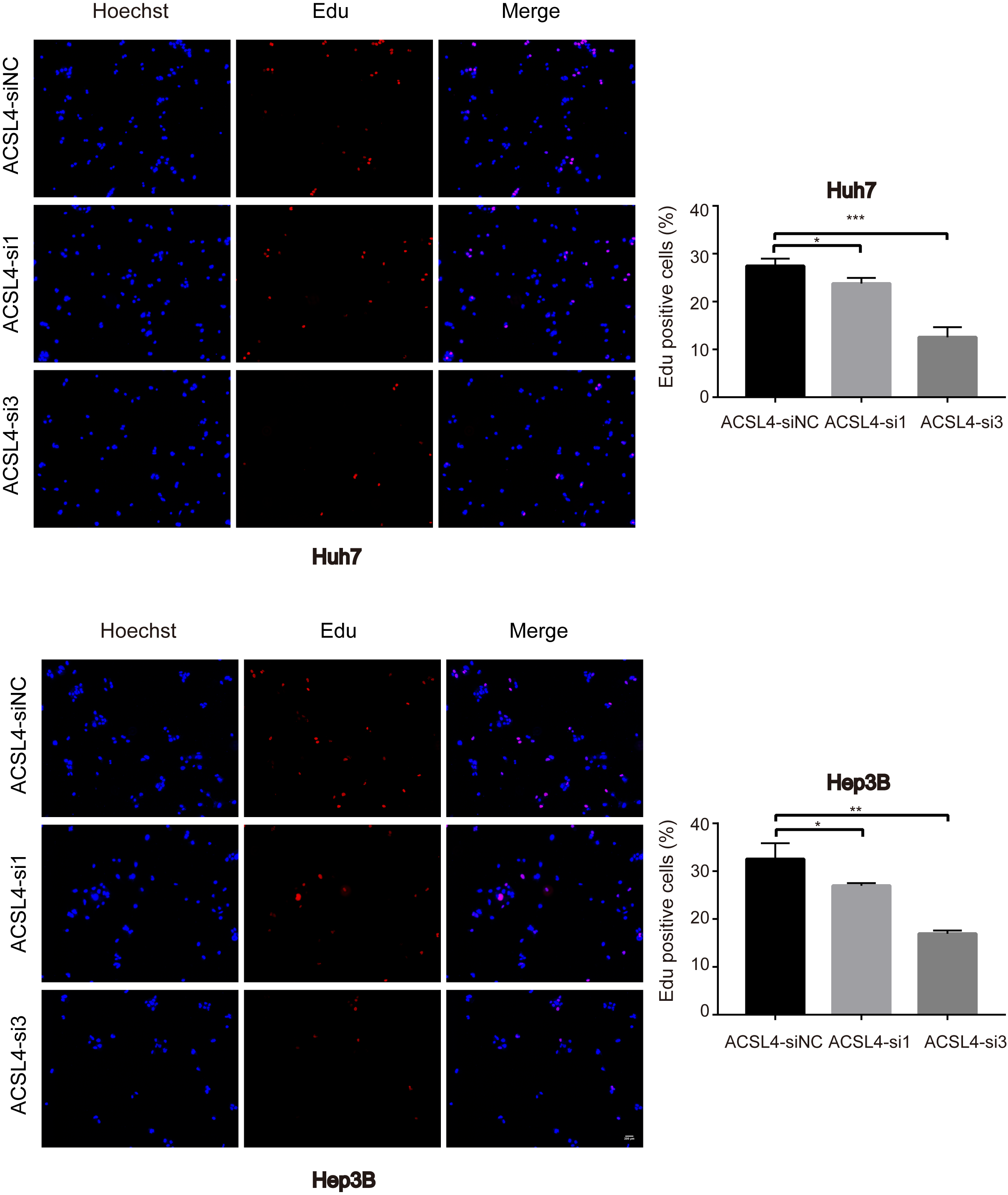

Supplement: Supplementary file 6 — Supplementary figure S2 [file 41389_2020_226_MOESM6_ESM.tif]

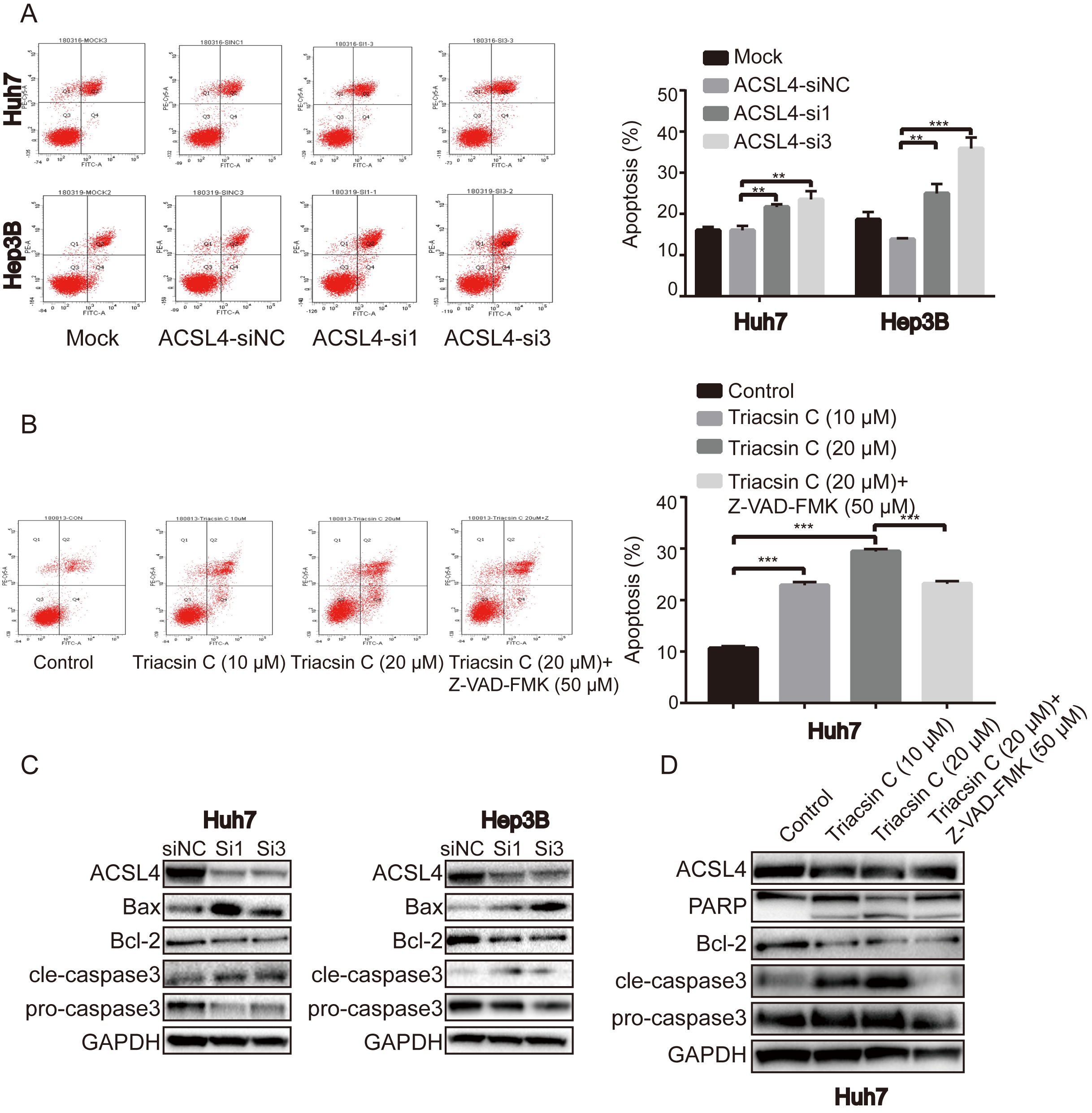

Supplement: Supplementary file 7 — Supplementary figure S3 [file 41389_2020_226_MOESM7_ESM.tif]

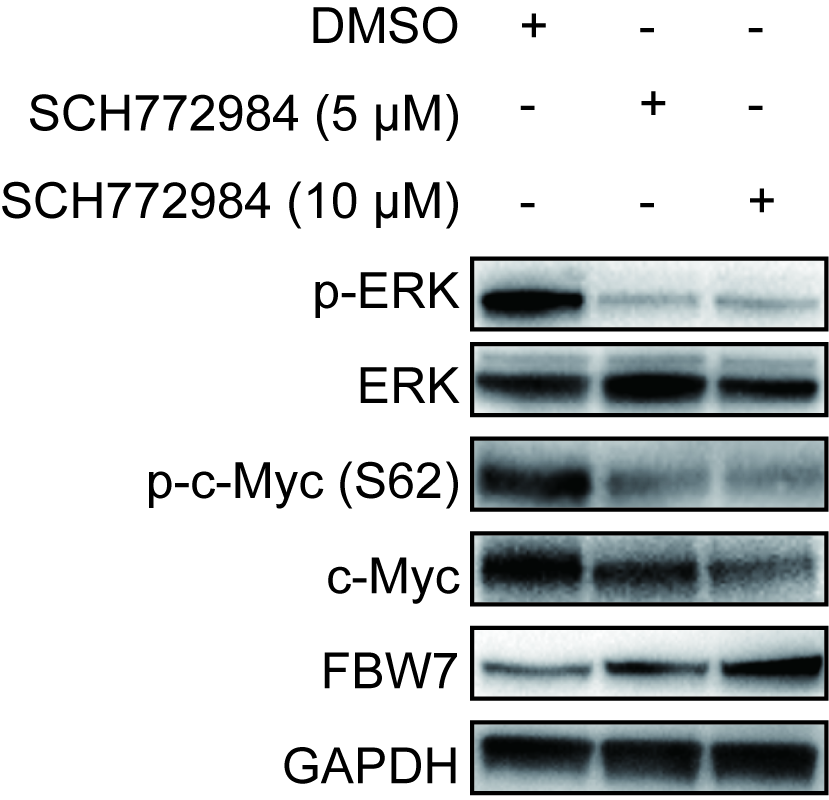

Supplement: Supplementary file 8 — Supplementary figure S4 [file 41389_2020_226_MOESM8_ESM.tif]
